# Poking Fun at Us

**Published:** 1871-03

**Authors:** 


### POKING FUN AT US.

The following was received in a letter from a correspondent in Germany. We will be glad to see Mrs. Simpson any day, and will present her with a pair of leather specs to enable her to see that her lamented husband did not read the book on " Health by Good Living " aright. By a more careful perusal of the chapter on " Biliousness," she will see that the statement was that nobody has any business to have bilious fevers, or rheumatics, or dyspepsia, or neuralgia ; but that if they will persist in bringing on these maladies, the quickest and very certain method of getting permanently cured is to ——— ; but the reader had better get the book, as some fifteen or twenty thousand sensible people have already done, to the great gratification of our worthy publishers, the Messrs. Hurd & Houghton, of 13 Astor Place, New York, and H. O. Houghton & Co., Boston; but now for the

PERMANENT AND EFFECTUAL CURE OF "BILIOUSNESS."

"Mr. Alexander Simpson, of Towanda, is dead. He was bilious, Mr. Alexander Simpson was, and he saw the following paragraph from the pen of Dr. Hall: —

"'If a bilious man wants to get well, and is in no special hurry, all that he has to do is to lie down out-of-doors, between two broad boards, and stay there until he gets ravenously hungry.'

"Mr. Simpson followed this advice, and calmly fell asleep with a broad board on top of him. Under ordinary circumstances there would have been no trouble; but there was a Fat Man's Ball in the lager-beer saloon, next door, that day, and the two champion fat men got over the fence, and sat down with a jerk on top of Mr. Alexander Simpson's upper board, without knowing he was there. It squelched the breath out of him at the first blow. And the fat men, you understand, they sat and sat there, and discussed politics, and the Alabama claims, and the Legal Tender Act, and the weather, and women's rights, and the Harrison boiler, and metaphysics, and they kept on drinking glass after glass of beer, and getting heavier and heavier, until one of them happened to look under the board, — and there was Alexander Simpson, as dead as Nebuchadnezzar, and mashed out so thin that you could pass him in under a closed door without scraping his vest buttons! He does not suffer from bile now. But does anybody know where Dr. Hall lives? Because Mrs. Simpson is making inquiries, and she is anxious to snatch a few silver hairs from his brow, and to necessitate the purchase of a patent glass eye."

